# JWA regulates TRAIL-induced apoptosis via MARCH8-mediated DR4 ubiquitination in cisplatin-resistant gastric cancer cells

**DOI:** 10.1038/oncsis.2017.57

**Published:** 2017-07-03

**Authors:** Q Wang, Q Chen, L Zhu, M Chen, W Xu, S Panday, Z Wang, A Li, O D Røe, R Chen, S Wang, R Zhang, J Zhou

**Affiliations:** 1 Department of Molecular Cell Biology and Toxicology, Key Laboratory of Modern Toxicology of Ministry of Education, School of Public Health, Nanjing Medical University, Nanjing, China; 2 Jiangsu Key Lab of Cancer Biomarkers, Prevention and Treatment, School of Public Health, Nanjing Medical University, Nanjing, China; 3 Laboratory of Cancer Biology, Biomedical Research Center, Sir Runrun Shaw Hospital, Zhejiang University, Hangzhou, China; 4 Department of Cancer Research and Molecular Medicine, Norwegian University of Science and Technology (NTNU), Trondheim, Norway; 5 Cancer Clinic, Department of Surgery, Levanger Hospital, Nord-Trøndelag Hospital Trust, Levanger, Norway; 6 Key Laboratory of Environmental Medicine Engineering, Ministry of Education, School of Public Health, Southeast University, Nanjing, China; 7 Department of Pharmaceutical Sciences, School of Pharmacy, Texas Tech University Health Sciences Center, Amarillo, TX, USA

## Abstract

Platinum chemotherapeutics are widely used to treat solid malignant tumors, including gastric cancer (GC). Drug resistance to platinum compounds may result in cancer relapse and decreased survival. The identification and development of novel agents to reactivate apoptosis pathways in platinum-resistant cancer cells is therefore necessary. Here we report that cisplatin-resistant human GC cells (BGC823/DDP and SGC7901/DDP) but not their parental cells (BGC823 and SGC7901) exhibit high sensitivity to tumor necrosis factor-related apoptosis-inducing ligand (TRAIL) as a result of overexpression of death receptor 4 (DR4). Furthermore, we found that JWA, a molecule that promotes cisplatin-induced apoptosis in GC cells, suppressed TRAIL-induced apoptosis via negative regulation of DR4. Mechanistically, JWA promoted the ubiquitination of DR4 at K273 via upregulation of the ubiquitin ligase membrane-associated RING-CH-8 (MARCH8). In human GC tissues, JWA and DR4 protein levels were negatively correlated. Thus TRAIL may serve as an auxiliary treatment for cisplatin-resistant GC, and JWA may be a potential predictive marker of TRAIL sensitivity and may improve personalized therapeutics for treating human GC.

## Introduction

Gastric cancer (GC) is the third leading cause of death resulting from cancer worldwide.^1, 2, 3^ Almost 50% of all GC cases are diagnosed in China.^1, 4^ Platinum-based chemotherapeutics remain the cornerstone of therapy for cancers, including GC.^5, 6^ However, innate or acquired resistance to platinum is very common in the treatment of solid tumors,^7^ and resistance to platinum agents, such as cisplatin (DDP), may be accompanied by cross-tolerance to DNA-damaging drugs, for example, gemcitabine, etoposide and 5-fluorouracil.^5^ Consistently with this observation, we have recently found that cisplatin-resistant GC cells exhibit a robust ability to repair DNA and are cross-resistant to DNA-damaging agents, such as arsenic trioxide (As_2_O_3_).^8, 9^ Platinum-based drugs primarily initiate an intrinsic apoptotic pathway through DNA damage response activation via the mitochondria apoptotic pathway.^10, 11^ The development of novel agents that reactivate other apoptosis pathways in platinum-resistant cancer cells might be a promising therapeutic strategy.

Beyond the mitochondrial apoptotic pathway, the death receptor (DR) pathway triggers apoptotic cell death and is involved in classical ligand–cell surface receptor interactions.^12^ Targeting the DR pathway has been proposed for future cancer therapies.^13^ Three types of DRs have been identified: tumor necrosis factor receptor 1 (TNFR1), TNF-related apoptosis-inducing ligand receptors (TRAIL-Rs, DR4/DR5), and Fas (also known as CD95 or Apo1).^12^ After binding to their respective ligands, DRs recruit Fas-associated death domain adaptor protein and activate death caspases, thereby causing apoptosis of the cell within hours.^14, 15^ DR ligands, such as purified TNF-α,^16^ Fas Ligand fusion proteins^17^ and recombinant soluble TRAIL,^18^ have demonstrated clinical potential for cancer therapy.

The clinical utilization of both TNF and FasL has been hampered by their toxic side effects.^14^ However, the unique ability of TRAIL to selectively eradicate cancer cells without affecting normal cells makes it an attractive potential treatment for cancer.^18^ Recombinant human TRAIL and DR4/DR5 antibodies have already been tested in clinical trials.^19^ TRAIL-induced apoptosis is achieved through caspase-8 activation; then, it directly activates caspase-3 and apoptosis or alternatively the apoptotic signal may be amplified through cleavage of the BH3-only protein Bid and the mitochondrial apoptotic signal pathway.^20, 21^ Several studies have revealed that drug resistance can be prevented or reversed by a combination of chemical drugs with TRAIL in cancer cell lines.^22, 23^ Interestingly, it has also been reported that chemo-resistant colon cancer side-population cells exhibit more sensitivity to TRAIL than do non-side population cells.^24^ In addition, the cisplatin-dependent upregulation of DRs (such as DR4 and DR5) increases TRAIL-induced apoptosis in esophageal squamous cell carcinoma.^25^ However, the regulatory mechanisms and effects related to TRAIL in GC cells resistant to cisplatin have not been clearly defined.

The JWA gene (ARL6IP5) is involved in cellular responses, such as heat shock and oxidative stress.^26, 27, 28^ This protein also promotes apoptosis induced by As_2_O_3_ via both the reactive oxygen species- and mitochondrial-linked pathway and p38 mitogen-activated protein kinase-linked tubulin polymerization in cell lines (HeLa, MCF-7).^29, 30^ Recently, we have demonstrated that JWA via the CK2/pXRCC1/XRCC1 axis promotes cisplatin-induced apoptosis in resistant human GC cells.^9^ In addition, JWA negatively regulates HER2 and activates extracellular signal–regulated kinase phosphorylation, thus leading to lapatinib resistance in human GC cells.^31^ Therefore, JWA may function as a regulator of chemotherapeutic-induced apoptosis. However, whether JWA is involved in TRAIL-triggered cell apoptosis in GC cells remains unclear.

Here we found that cisplatin-resistant human GC cells were significantly more sensitive to TRAIL with concomitant elevated DR4 expression than their parental cisplatin-sensitive GC cells. Mechanistically, JWA promoted the ubiquitination of DR4 at K273 via the upregulation of the ubiquitin ligase membrane-associated RING-CH-8 (MARCH8) and decreased TRAIL sensitivity in human GC cells. Our work highlights that (1) TRAIL effectively induces apoptosis in cisplatin-resistant GC cells; (2) the JWA/MARCH8/DR4 axis controls the effect of TRAIL on cisplatin-resistant human GC cells; and (3) JWA is a potential predictive marker of TRAIL sensitivity in GC, thus indicating its significance in clinical personalized therapeutics.

## Results

### Acquired cisplatin-resistant human GC cells are sensitive to TRAIL

**Figure 1:**
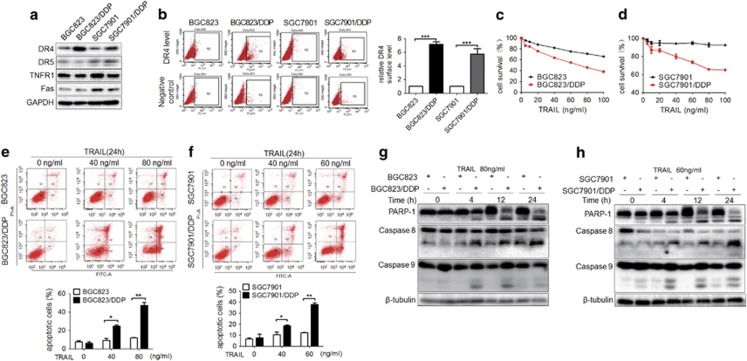
Acquired cisplatin-resistant human GC cells are sensitive to TRAIL. (**a**) The expression of DR4, DR5, TNFR1 and Fas in human GC cells BGC823 and SGC7901 and their cisplatin-resistant variants was determined by western blotting. (**b**) Left: Cell surface DR4 expression in BGC823 and SGC7901 cells and their variants was analyzed by flow cytometry. Right: Comparison of relative DR4 surface expression between different groups. Respective DR4 surface expression was estimated by examining the difference between the values (percentage of positive cells) of PE-DR4 and its corresponding IgG-PE control from at least three experiments in the same group. After 24 h of TRAIL treatments at the indicated concentrations in (**c**) BGC823 and its variant and (**d**) SGC7901 and its variant, the cell viability was determined by Cell Counting Kit-8 (CCK8) assays. After 24 h of TRAIL treatments at the indicated concentrations in (**e**) BGC823 and its variant and in (**f**) SGC7901 and its variant, cell apoptosis was determined by flow cytometry, and the quantification of apoptotic cells is indicated below. After exposure to TRAIL at the indicated concentrations and time points, the cleaved-PARP-1 and cleaved-caspase-8 and -9 levels were determined by western blotting analyses in BGC823 and its variant (**g**) or SGC7901 and its variant (**h**). The experiments were independently performed at least three times, and representative data are shown. For flow cytometry and CCK8 assays, the results are shown as the means±s.e.m., *P<0.05, **P<0.01, ***P<0.001.

To investigate whether DRs are involved in cisplatin resistance in human GC cells, DR expression patterns (DR4/DR5, TNFR1 and Fas) in two human GC cell lines (BGC823, SGC7901) and their cisplatin-resistant variants were analyzed. Only DR4 was significantly upregulated in the cisplatin-resistant variants (Figure 1a). The upregulated DR4 expression was also measured by flow cytometry in both cisplatin-resistant variants (Figure 1b). These results indicated that cisplatin-resistant GC cells may be sensitive to TRAIL, because DR4 is a DR for TRAIL. To test this possibility, the GC cells were exposed to TRAIL at various concentrations for 24 h. Cell Counting Kit-8 (CCK8) assays showed that both cisplatin-resistant variants were more sensitive to TRAIL than were their parental GC cells (Figures 1c and d). Flow cytometry also indicated more apoptosis in two cisplatin-resistant variants than in their parental GC cells after TRAIL treatment for 24 h (Figures 1e and f). Western blotting analyses indicated that the cisplatin-resistant GC cells expressed higher levels of cleaved-caspase-8 and -PARP-1 (poly ADP-ribose polymerase-1) than did BGC823 and SGC7901 cells after TRAIL treatment, and this effect showed a time-dependent pattern (Figures 1g and h). Cleaved-caspase-9 was also increased in both BGC823/DDP and SGC7901/DDP cells, compared with BGC823 and SGC7901 cells, and this effect was not time dependent (Figures 1g and h).

### GC cells are sensitized to TRAIL by DR4

**Figure 2:**
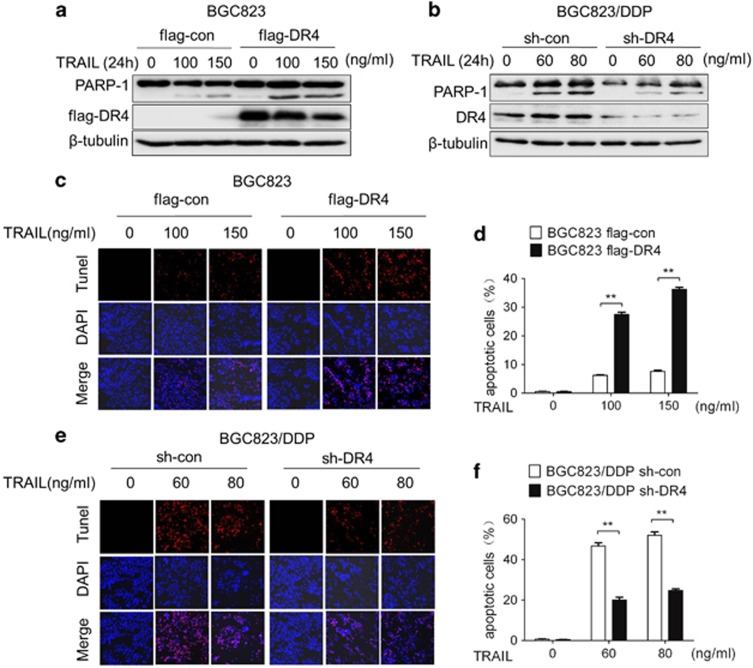
GC cells are sensitized to TRAIL by DR4. Flag-DR4-transfected BGC823 cells (**a**) and sh-DR4-transfected BGC823/DDP cells (**b**) were exposed for 24 h to the indicated TRAIL concentrations and cleaved-PARP-1, and DR4 was then determined by western blotting analyses. Apoptosis was determined by TUNEL assay after exposure to the indicated TRAIL concentrations for 24 h in Flag-DR4-transfected BGC823 cells (**c**) and sh-DR4-transfected BGC823/DDP cells (**e**); the quantitative apoptosis ratios of (**c**, **e**) are shown in (**d**, **f**), respectively. The experiments were independently performed at least three times, and representative data are shown. For the TUNEL assay, the data are expressed as the means±s.e.m., **P<0.01.

To determine the contribution of DR4 in sensitizing GC cells to TRAIL, DR4 was knocked down or overexpressed in GC cells and was followed by TRAIL treatment. As shown in Figure 2a, the cleaved-PARP-1 levels increased in DR4-overexpressing BGC823 cells. In contrast, the levels of cleaved-PARP-1 decreased in DR4-knockdown BGC823/DDP cells (Figure 2b).

TUNEL assays further confirmed that the apoptosis rate in DR4-overexpressing BGC823 cells increased after TRAIL treatment (Figures 2c and d). In contrast, decreased apoptosis was observed in DR4-knockdown BGC823/DDP cells (Figures 2e and f). To verify whether DR5 was involved in cisplatin resistance, si-DR5-2 was used to knock down DR5 in BGC823/DDP cells (Supplementary Figure S1a). In contrast to the results after DR4 knockdown, cleaved-caspase-3 was slightly decreased after TRAIL treatment in DR5-knockdown BGC823/DDP cells (Supplementary Figure S1b). The results revealed that DR4 but not DR5 is an important player in controlling the sensitivity of GC cells to TRAIL.

### JWA negatively regulates DR4 expression and TRAIL sensitivity in GC cells

**Figure 3:**
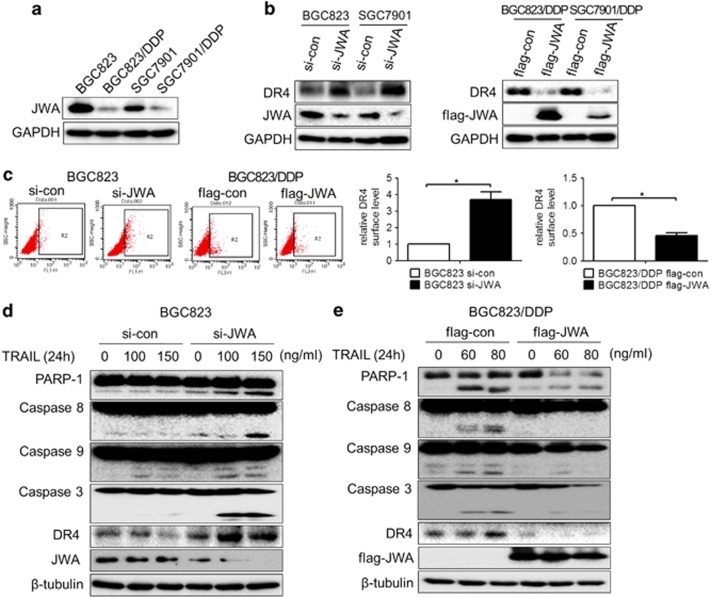
JWA negatively regulates DR4 expression and sensitivity of GC cells to TRAIL. (**a**) JWA expression in GC cells BGC823 and SGC7901 and their variants was determined by western blotting analyses. (**b**) GC cells BGC823 and SGC7901 and their variants were transfected with si-JWA or Flag-JWA or a corresponding control for 48 h; DR4 and JWA expression in these cells was determined by western blotting analyses. (**c**) Left: Si-JWA or Flag-JWA GC cells or a corresponding control were transfected into BGC823 or its variant for 48 h; the DR4 surface expression was then analyzed by flow cytometry. Right: Comparison of relative DR4 surface expression between different groups. Respective DR4 surface expression was estimated as depicted in Figure 1b. Si-JWA transfected-BGC823 (**d**) and Flag-JWA transfected-BGC823/DDP cells (**e**) were exposed to the indicated TRAIL concentrations for another 24 h; JWA and DR4 expression and the apoptotic biomarkers cleaved-PARP-1 and cleaved-caspase-8, -9 and -3 were determined by western blotting analyses. The experiments were independently performed at least three times, and representative data are shown. For the flow cytometric assay, the data are shown as the means±s.e.m., *P<0.05.

**Figure 4:**
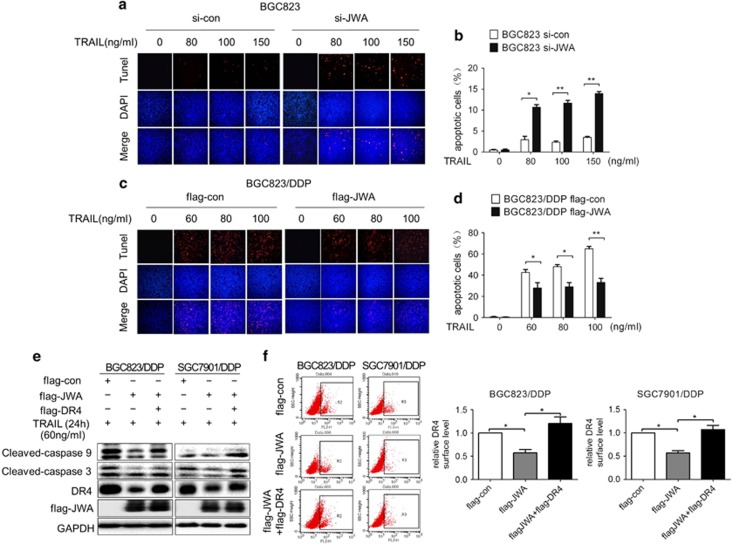
JWA via DR4 inhibits TRAIL-induced apoptosis in GC cells. The apoptosis of si-JWA-transfected BGC823 cells (**a**) and Flag-JWA-transfected BGC823/DDP cells (**c**) was determined by TUNEL assays after exposure to the indicated TRAIL concentrations for 24 h; quantitative apoptosis ratios of (**a**, **c**) are shown in (**b**, **d**), respectively. Flag-con or Flag-JWA, or Flag-JWA plus Flag-DR4 plasmids were transfected into GC cells BGC823/DDP and SGC7901/DDP for 48 h and then exposed to 60 ng/ml TRAIL for an additional 24 h. The levels of Flag-JWA, DR4 and cleaved-caspase-9 and -3 were determined by western blotting analyses (**e**), and DR4 surface expression was analyzed by flow cytometry and by the quantification and comparison of relative DR4 surface expression among different groups, as depicted in Figure 1b (**f**). The experiments were independently performed at least three times, and representative data are shown. For the TUNEL and flow cytometric assays, the data are expressed as the means±s.e.m., *P<0.05, **P<0.01.

Endogenous JWA expression was lower in both cisplatin-resistant variants than in their parental cells (Figure 3a). To investigate whether DR4 was regulated by JWA, si-JWA or Flag-JWA was used to knock down JWA in BGC823/SGC7901 or to overexpress JWA in BGC823/DDP/SGC7901/DDP cells, respectively. The results showed that DR4 expression was negatively regulated by JWA in these GC cells (Figure 3b). The flow cytometric analyses also confirmed the above results (Figure 3c and Supplementary Figure S2a). Moreover, knocking down JWA expression in BGC823 not only increased the levels of DR4 but also increased the TRAIL-induced expression of cleaved-caspase-8, -9 and -3 and cleaved-PARP-1 (Figure 3d). In contrast, transfection of Flag-JWA not only inhibited DR4 expression but also suppressed the effect of TRAIL-induced apoptosis, and we observed decreased levels of cleaved-caspase-8, -9 and -3 and cleaved-PARP-1 in BGC823/DDP cells (Figure 3e). Accordingly, a TUNEL assay indicated that the apoptotic rate increased in JWA-knockdown BGC823 cells (Figures 4a and b), and decreased in JWA-overexpressing BGC823/DDP cells (Figures 4c and d) after TRAIL treatment for 24 h.

To determine whether JWA modulated the sensitivity of GC cells to TRAIL through DR4, Flag-JWA and Flag-DR4 were cotransfected into BGC823/DDP and SGC7901/DDP cells, and cells were then treated with TRAIL for 24 h. The data showed that the decreased proapoptotic effects of TRAIL induced by JWA overexpression (lanes 2 and 5) were recovered by Flag-DR4 (lanes 3 and 6) (Figure 4e). The flow cytometric analyses further confirmed that DR4 expression in Flag-DR4-transfected BGC823/DDP and SGC7901/DDP cells was increased (Figure 4f). These results indicated that DR4 is required for JWA to regulate the sensitivity of GC cells to TRAIL-induced apoptosis.

### JWA inhibits the DR4 expression by promoting its lysosomal degradation

**Figure 5:**
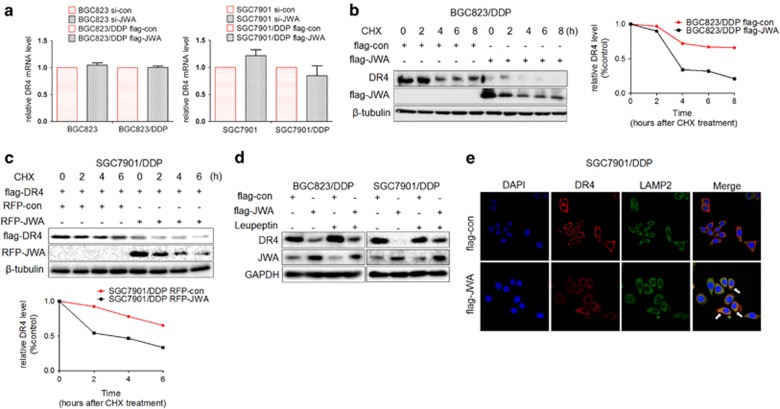
JWA inhibits DR4 expression by promoting its lysosomal degradation. (**a**) GC cells BGC823 and SGC7901 or their variants were transfected with si-JWA or Flag-JWA or a corresponding control for 48 h, and the DR4 mRNA in these cells was analyzed by reverse transcriptase–PCR (RT–PCR). (**b**) Left: Flag-JWA or Flag-con was transfected into GC cells BGC823/DDP for 48 h, and cells were then exposed to cycloheximide (CHX) at 50 μg/ml for the indicated times. DR4 protein stability was determined by western blotting. Right: Quantification curve of the DR4 protein level in BGC823/DDP cells. (**c**) Above: RFP-JWA and Flag-DR4 were cotransfected into SGC7901/DDP GC cells for 48 h, and cells were then exposed to 50 μg/ml CHX for the indicated times. Flag-DR4 protein stability was determined by western blotting. Below: Quantification curve of the Flag-DR4 protein level in SGC7901/DDP cells. (**d**) Flag-JWA or a corresponding control was transfected into cisplatin-resistant GC cells (SGC7901/DDP, BGC823/DDP) for 36 h; the cells were then exposed to another 20 h of leupeptin (5 μM), and the DR4 levels were determined by western blotting. (**e**) SGC7901/DDP GC cells were transfected with Flag-con or Flag-JWA for 48 h. A representative picture shows DR4 (red), the lysosome marker Lamp2 (green), the 4,6-diamidino-2-phenylindole-labeled nucleus (blue) and the colocalization of the three signals (merge), as determined by immunofluorescence assays. The experiments were independently performed at least three times, and representative data are shown. For the quantitative RT–PCR assay, the data are presented as the means±s.e.m.; NS, no significance.

How does JWA negatively regulate DR4 in GC cells? To determine the possible mechanism, DR4 mRNA and protein levels were analyzed in JWA-knockdown and JWA-overexpressing GC cells. The DR4 mRNA levels were not markedly changed in JWA-deficient or -overexpressing GC cells (Figure 5a). However, accelerated degradation of DR4 was observed in JWA-overexpressing BGC823/DDP cells after cycloheximide treatment (Figure 5b). Moreover, exogenous DR4 was more rapidly degraded in JWA-overexpressing SGC7901/DDP cells than in control cells (Figure 5c). Because DR4 is turned over via ubiquitin-dependent routing to lysosomes,^32, 33^ we also analyzed whether the effect of JWA was associated with the lysosome pathway. After leupeptin treatment, decreased DR4 expression due to JWA overexpression was reversed in BGC823/DDP and SGC7901/DDP cells (Figure 5d). Moreover, immunofluorescence assays showed that DR4 (red) translocated from the plasma membrane to the cytoplasm and colocalized with the lysosome marker Lamp2 (green) in JWA-overexpressing SGC7901/DDP cells (Figure 5e). Overall, the data indicated that JWA negatively regulates DR4 expression via the ubiquitin-dependent lysosome pathway, which mediates the sensitivity of cisplatin-resistant GC cells to TRAIL.

### JWA promotes the ubiquitination of DR4 at the K273 site by increasing the expression of MARCH8

**Figure 6:**
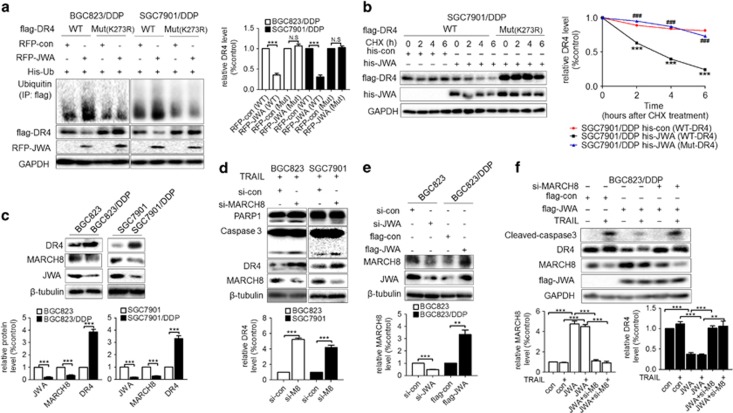
JWA promotes ubiquitination of DR4 at K273 via upregulation of MARCH8 in GC cells. (**a**) Left: GC cells (BGC823/DDP, SGC7901/DDP) were cotransfected RFP-JWA and His-Ub with Flag-DR4 (wild type (WT)) or Flag-DR4 (mutant K273R) for 48 h. Immunoprecipitation was used to determine the ubiquitination of DR4; and the expression of ubiquitin, Flag-DR4 and RFP-JWA was determined by western blotting analyses. Right: Quantification of the Flag-DR4 protein level in the two GC cell lines. (**b**) Left: GC cells SGC7901/DDP were cotransfected with his-JWA and Flag-DR4 (WT) or Flag-DR4 (Mut K273R) for 48 h and then were treated with cycloheximide (CHX) at 50 μg/ml for the indicated times, and the Flag-DR4 protein stability was determined by western blotting analyses. Right: Quantification curves of the Flag-DR4 protein level in SGC7901/DDP cells. His-con (WT-DR4) vs His-JWA (WT-DR4), ***P<0.001; His-JWA (Mut-DR4) vs His-JWA (WT-DR4), ^###^*P*<0.001 (**c**) Above: The expression of DR4, MARCH8 and JWA in GC cells BGC823 and SGC7901 and their variants were determined by western blotting analyses. Below: Quantifications of the JWA, MARCH8 and DR4 protein level in the GC cells. (**d**) Above: Si-MARCH8 or its control was transfected into GC cells BGC823 and SGC7901 for 48 h and then exposed to TRAIL (BGC823: 150 ng/ml, SGC7901: 100 ng/ml) for an additional 24 h. Western blotting analyses determined the expression of MARCH8, DR4, cleaved-caspase-3 and PARP-1. Below: Quantification of DR4 protein level in the two GC cell lines. (**e**) Above: BGC823 cells were transfected with si-JWA or BGC823/DDP cells were transfected with Flag-JWA for 48 h. The expression of MARCH8 and JWA was then determined by western blotting analyses. Below: Quantifications of MARCH8 protein level in the two GC cell lines. (**f**) Above: Flag-con, Flag-JWA or Flag-JWA with MARCH8 siRNA were transfected into BGC823/DDP cells for 48 h, and the cells were then exposed to TRAIL at 80 ng/ml for another 24 h. Western blotting analyses determined the expression of Flag-JWA, MARCH8, DR4 and cleaved-caspase-3. Below: Quantification of MARCH8 and DR4 protein level in BGC823/DDP cells. The experiments were independently performed at least three times, and representative data are shown. **P<0.01, ***P<0.001; NS, no significance.

Lysine 273 is critical for the ubiquitination-related stability of DR4.^32^ To confirm whether JWA modulates DR4 degradation through a similar mechanism, RFP-JWA and His-Ub were cotransfected with Flag-DR4 wild-type or Flag-DR4 mutant (K273R) into the two cisplatin-resistant GC variants; the results indicated that JWA promoted the degradation of wild-type DR4 but not the K273 mutant (Figure 6a). These results were verified in a cycloheximide chase experiment: exogenous DR4 (Mut K273R) was much more stable than its wild type in the JWA-overexpressing BGC823/DDP and SGC7901/DDP cells (Figure 6b and Supplementary Figure S3a). The flow cytometric analyses showed that upregulation of JWA did not affect Mut K273R DR4 expression (Supplementary Figure S3b), thus suggesting that JWA promoted DR4 ubiquitination at the K273 site.

Because MARCH8 has been reported to act as an E3 ligase mediating DR4 K273 ubiquitination,^32^ we further determined whether JWA modulates DR4 stability via a MARCH8-related pathway in GC cells. MARCH8 showed greater downregulation in the two cisplatin-resistant GC variants than in their respective parental GC cells, thus indicating a positive correlation between MARCH8 and JWA. In contrast, a negative correlation between MARCH8 and DR4 was observed (Figure 6c). In addition, knocking down MARCH8 in BGC823 and SGC7901 cells resulted in the upregulation of DR4 and increased the expression of cleaved-PARP-1 and -caspase-3 after TRAIL treatment, thus suggesting that MARCH8 negatively regulated DR4 and decreased TRAIL-mediated apoptosis (Figure 6d). Moreover, JWA was found to positively regulate the expression of MARCH8 in GC cells (Figure 6e). Furthermore, knocking down MARCH8 increased cleaved-caspase-3 levels in JWA-overexpressing BGC823/DDP cells after treatment with TRAIL, and JWA was unable to adequately downregulate DR4 when MARCH8 was knocked down in GC cells (Figure 6f). These results indicated that the JWA’s function in promoting the ubiquitination of DR4 occurred via the positive regulation of MARCH8 in GC cells.

### Human GC tissues confirm a negative correlation between JWA and DR4

**Figure 7:**
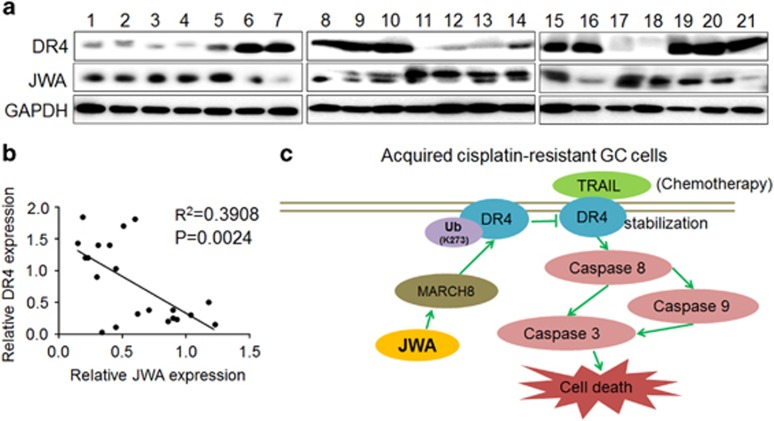
Protein levels of JWA and DR4 in human GC tissues. (**a**) Fresh human GC tissues from 21 patients were analyzed for JWA and DR4 protein expression by western blotting analyses. The loading control was GAPDH. (**b**) Correlation analysis between JWA and DR4 protein expression in the 21 GC tissues from (**a**). (**c**) Diagram illustrating the proposed molecular mechanism of how JWA is involved in DR4 degradation and decreases TRAIL-induced apoptosis in cisplatin-resistant GC cells. (1) In acquired cisplatin-resistant GC cells, substantially inhibited JWA expression results in the suppression of MARCH8. (2) Accordingly, suppressed MARCH8 further decreases ubiquitination of DR4 at K273. (3) The decreased DR4 ubiquitination confers greater stability in GC cells. (4) As a result, the DR4-based proapoptotic effect of TRAIL is dramatically enhanced, owing to the lower JWA expression in cisplatin-resistant GC cells.

To confirm whether the correlation between JWA and DR4 also existed in human GC tissues, western blotting analyses were used to assess JWA and DR4 protein levels in 21 random snap-frozen surgical GC samples. As shown in Figures 7a and b, although both JWA and DR4 displayed clearly heterogeneous expression patterns, the expression intensity of JWA and DR4 indicated a significant negative correlation between the two (*R*^2^=0.3908, *P*=0.0024).

## Discussion

This study demonstrates that TRAIL may be a useful agent inducing cell apoptosis in cisplatin-resistant human GC cells and that JWA functions as an upstream regulator that promotes the ubiquitination of DR4 at K273 via the upregulation of the ubiquitin ligase MARCH8, thus indicating that JWA is a potential predictive marker for TRAIL sensitivity that may improve personalized therapeutics for human GC.

Cisplatin is widely used in regimens for GC therapy.^34^ However, even patients who respond to cisplatin treatment ultimately develop acquired resistance.^35^ Drug resistance development is multifactorial, and disordered cell signaling networks may be important in resistance.^36, 37^ Cell apoptosis induced by cisplatin is in part achieved via mitochondrial-associated apoptotic pathways.^5, 10^ The development of new non-mitochondrial apoptotic pathway agents is needed because they may reactivate apoptosis in cisplatin-resistant cells. The DR pathway is an extrinsic apoptotic pathway in which cell surface DRs transmit apoptotic signals initiated by specific death ligands.^12, 13, 21^

From the perspective of the DR pathway, we found that the levels of DR4 (the receptor of TRAIL) were significantly elevated in the cisplatin-resistant variants compared with their parental cisplatin-sensitive cells, thus suggesting that cisplatin-resistant human GC cells may be susceptible to TRAIL. Both recombinant human TRAIL and DR4/5 agonistic antibodies have shown encouraging antitumor activities in clinical trials.^38^ Numerous reports have demonstrated a synergy between TRAIL and cisplatin in different types of cancer cells.^39, 40^ Here we provide the first demonstration that human GC cells with acquired cisplatin resistance are significantly more sensitive to TRAIL than their parental cells owing to the former’s high DR4 expression.

In the present study, cleaved-PARP-1 and -caspase-8 were found to increase in a time-dependent manner after TRAIL treatment, whereas cleaved-caspase-9 did not show similar time course changes, although it was also increased at an early stage (4 h). Other studies have shown that the TRAIL signaling pathway can activate both the DR pathway and mitochondrial apoptosis pathway (through Bid cleavage) in some cancer cells; however, the enhanced expression of Bcl2 inhibits Bid and subsequently inhibits caspase-9 activation.^20^ Our previous study shows that Bcl2 expression is higher in both BGC823/DDP and SGC7901/DDP GC cells than in their parent GC cells, that is, BGC823 and SGC7901.^41^ Compared with that in the cisplatin-sensitive GC cells, Bid expression was higher in the cisplatin-resistant variants (data not shown). Therefore, TRAIL treatment activated both the DR and mitochondrial pathway at an early stage in resistant GC cells. Later, the action of Bid on caspase-9 might be inhibited by Bcl2. As a result, unlike cleaved-caspase-8, which was expressed in a time-dependent manner, cleaved-caspase-9 showed a cleavage peak only in the early stage; however, the exact molecular mechanisms still require clarification.

To confirm that TRAIL sensitivity is mediated by DR4, we provided evidence that increasing the expression of DR4 alone in cisplatin-sensitive GC cells enhanced their TRAIL sensitivity and cell apoptosis. In contrast, knocking down DR4 expression in the cisplatin-resistant variants decreased TRAIL sensitivity and TRAIL-triggered apoptosis.

In our previous studies, JWA was identified as an important factor for chemotherapy-induced apoptosis.^30, 42^ JWA enhances cisplatin sensitivity in acquired cisplatin-resistant human GC cells (BGC823/DDP, SGC7901/DDP).^9^ The results led us to investigate whether JWA might have an effect on apoptosis in GC cells induced by TRAIL. Here we provide new evidence that JWA negatively regulates DR4 expression in GC cells. The role of JWA in DR4-mediated apoptosis was hypothesized because DR4/5 has been shown to activate caspase-8 and to activate caspase-3 either directly or via caspase-9, thus ultimately resulting in apoptosis.^7^ To confirm this hypothesis, we knocked down JWA expression in BGC823 cells and found increased levels of DR4 and the activation of cleaved-caspase-8, -9 and -3 by TRAIL. In contrast, in BGC823/DDP GC cells, overexpression of JWA resulted in decreased DR4 and blocked TRAIL-induced activation of these caspases. This new evidence contrasted with the pro-apoptotic role of JWA that we previously identified and showed that JWA counteracts TRAIL-induced apoptosis by downregulating DR4 expression.

Most cell surface receptors are recycled through the lysosome pathway.^33^ DR4, as a cell surface receptor, is recycled or degraded via an internalization-linked mechanism.^32, 33^ A recent study has shown that DR4 is degraded via ubiquitin-dependent routing to lysosomes.^32^ Our results showed that JWA did not transcriptionally regulate DR4 in GC cells. However, JWA overexpression induced the rapid degradation of DR4 via the ubiquitin-dependent lysosome pathway. The negative correlation between JWA and DR4 was also confirmed in human snap-frozen GC tumor tissues. Therefore, JWA is an important sensor that controls the expression level of DR4 via ubiquitin-dependent lysosomal degradation in GC cells.

The ubiquitin acceptor site K273 in DR4 is crucial for DR4 ubiquitination.^32^ Here we confirmed that K273 in DR4 is a target site of JWA-induced ubiquitination in GC cells. The MARCH ubiquitin ligase family has a role in degrading membrane molecules, and MARCH8 is an E3 ligase that ubiquitinates DR4 for subsequent lysosomal degradation.^32^ Our results indicated that JWA induced ubiquitin-dependent lysosomal degradation of DR4 via the upregulation of MARCH8 expression, thereby decreasing the sensitivity of GC cells to TRAIL. Therefore, in acquired cisplatin-resistant GC cells, the decreased expression of JWA resulted in the downregulation of MARCH8 expression. Accordingly, the decreased expression of MARCH8 decreased the ubiquitination of DR4 at K273, thereby conferring more stable DR4 expression. As a result, a higher level of DR4 enhanced the sensitivity of cisplatin-resistant GC cells to TRAIL (Figure 7c).

GTRAP3-18, a homolog of JWA in rats, regulates intracellular trafficking events^43^ by binding Rab1 and consequently inhibiting the endoplasmic reticulum-to-Golgi trafficking of Rab1.^44^ GTRAP3-18 has been also identified as an interaction partner for the glutamate transporter EAAC1 at the plasma membrane and found to negatively and dominantly regulate cellular glutathione content.^45, 46^ The action of JWA on the MARCH8 levels might be associated with its intracellular trafficking role. JWA may directly bind to MARCH8 or indirectly bind to its interacting protein, thereby inhibiting endosome-to-lysosome trafficking of MARCH8; this inhibition may subsequently prevent MARCH8 degradation. Accordingly, accumulated MARCH8 may enhance the ubiquitin-mediated degradation of the substrate DR4. However, the molecular mechanisms remain to be elucidated.

The present study provides data supporting the important role of JWA as a sensor controlling TRAIL sensitivity in human GC cells. In the core mechanism, JWA promotes the ubiquitination of DR4 at K273 via the upregulation of MARCH8 expression. Our data indicated that TRAIL may serve as a potential treatment for GC resistant for cisplatin. JWA is a potential biomarker for predicting TRAIL sensitivity and may improve the development of personalized therapeutics and precision medicine for the treatment of human GC.

## Materials and methods

### Cell lines and growth conditions

The human GC cell lines BGC823 and SGC7901 and the cisplatin-resistant variants of these two cell lines were maintained as previously described.^8^ Short tandem repeat profiling and mycoplasma contamination were used to authenticate the source of the cell lines.^8^ Prior to the experiments, cisplatin-resistant variants were maintained without cisplatin for 14 days.

### GC tumor tissue specimens

Prior to this study, an institutional agreement was obtained from the Institutional review boards of Nanjing Medical University. Informed consent for collecting GC tumor tissues was also obtained from the GC patients. Human GC tumor tissues were obtained from the teaching hospital of Nanjing Medical University, Yixing People’s Hospital (Yixing, Jiangsu, China).

### Plasmids, siRNA and cell transfection

The details of Flag-JWA and the control plasmids have been described in a previous study.^30^ RFP-JWA and his-JWA plasmids were constructed on the basis of the Flag-JWA plasmid. The Flag-DR4 wild-type or its mutant (K273R) constructs were inserted into the GV141 eukaryotic expression vector (Shanghai Genechem, Shanghai, China) using XhoI/KpnI sites. The DR4 siRNA (5′-TTGTAAATCAGATGAAGAA-3′) expression cassette was cloned into the GV248 vector to produce DR4 shRNA (Shanghai Genechem). The JWA (5′-CGAGCTATTTCCTTATCTC-3′), MARCH8 (5′-CCUUGUAUGUGCUCAUUGA-3′) and DR5-2 (5′-UUCUGGGAACACGAGCAACAG-3′) siRNAs and a nonspecific control siRNA were synthesized by Ribobio (Guangzhou, China). siRNA and plasmid DNA were transfected into GC cells with Lipofectamine2000 (Invitrogen, Grand Island, NY, USA) according to the manufacturer’s protocol.

### Cytotoxicity assay

Prior to TRAIL treatment, approximately 5000 cells were seeded per well in the 96-well plates, 12 h later, and TRAIL (Peprotech, Rocky Hill, NJ, USA) was used to treat the cells at the indicated concentrations for 24 h. A Cell Counting Kit-8 Kit (Dojindo, Kumamoto, Japan) was used to detect cell viability according to the protocol. Cell survival rates are shown as the mean±s.e.m. in the results.

### Apoptosis assay

GC cells were treated with the different concentrations of TRAIL for 24 h. Then a TUNEL Detection Kit (Roche, Basel, Switzerland) was used to determine cell apoptosis, according to the manufacturer’s protocol. Cell confocal images were acquired with a Zeiss LSM700 system (Carl Zeiss, Jena, Germany). Percentages of apoptotic cells are shown from at least 1000 cells, and data were obtained from three independent experiments.

### Flow cytometric assay

An Apoptosis Detection Kit (FITC A/V, BD, San Jose, CA, USA) was also used to detect apoptosis, according to the manufacturer’s instructions, by using flow cytometric analyses. After 24 h of treatment with different concentrations of TRAIL, the GC cells were collected and washed twice using phosphate-buffered saline. Then 5 μl of Annexin V and 5 μl of propidium iodide were added to 100 μl of binding buffer with the cells and incubated for 15 min at room temperature in a dark room. Subsequently, 300 μl of binding buffer was added to the mixture, which was then analyzed by flow cytometry (FACSCanto; BD) within 1 h.

### Western blotting assay

Western blotting analyses were carried out as previously reported.^29^ All antibodies in this study were used as follows: anti-β-tubulin (1:1000, Huaan, Hangzhou, China); anti-JWA (1:500, AbMax, Beijing, China); anti-GAPDH (anti-glyceraldehyde 3-phosphate dehydrogenase), anti-his, and anti-Flag (1:2000, Beyotime, Shanghai, China); anti-MARCH8 and anti-DR4 (1:1000, Proteintech, Chicago, IL, USA); anti-DR4 and anti-Ub (1:500, Santa Cruz, Dallas, TX, USA); anti-PARP-1 and anti-caspase-8, -9, -3 (1:1000, Cell Signaling Technology, Beverly, MA, USA); anti-RFP, anti-TNFR1, anti-Fas and anti-DR5 (1:2000, Abcam, Cambridge, UK). Cycloheximide (Sigma-Aldrich, St Louis, MO, USA) and Lseupeptin (Amresco, Solon, OH, USA) were used at the indicated concentrations.

### Immunofluorescence microscopic assay

The details of indirect immunofluorescence have been described previously.^30^ Briefly, the cells were incubated with a rabbit anti-Lamp2 antibody (1:300, Proteintech) and a mouse anti-DR4 antibody (1:300, Abcam, Cambridge, UK) for 12 h and then with corresponding Alexa Fluor-labeled secondary antibodies (Beyotime) at a 1:300 dilution for another 1 h. Sequentially, the cells were incubated with 4,6-diamidino-2-phenylindole (Beyotime, China) for 15 min. The results of cell confocal images were acquired with a LSM 700 system (Carl Zeiss).

### Immunoprecipitation analyses

The details of the immunoprecipitation experiments have been described in a previous report.^28^ Briefly, the treated cells were harvested and lysed in cold lysis buffer. Cell extracts were obtained by centrifugation at approximately 11 500 r.p.m. at 4 °C for 15 min (Allegra X-30R, BECKMAN, Brea, CA, USA). As a pretreatment, protein A/G agarose beads (Santa Cruz) were added into resuspended cell pellets to conjugate and remove nonspecific immune globulins. Then anti-Flag antibody (1:100, Beyotime) was incubated with the lysates for 1 h, and protein A/G agarose beads were added and incubated with the lysates overnight. The beads were collected and washed three times using lysis buffer, and loading buffer was added to the beads. Elution of the immunoprecipitates and western blotting analyses were subsequently performed.

### Real-time PCR analyses

The details of RNA extraction and reverse transcriptase–PCR processes were as described in a previous report.^9^ The following primers were used to amplify cDNA: GAPDH 5′-GGGAAGCTCACTGGCATGGCCTTCC-3′ (R) and 5′-CATGTGGGCCATGAGGTCCACCAC-3′ (F); and DR4 5′-GAAACACACCCTGTCCATGCACTT-3′ (R) and 5′-TGGCACACAGCAATGGGAACATAG-3′ (F).

### Cell surface DR4 expression analysis

Cell surface expression levels of DR4 were analyzed by flow cytometry (FACSCanto; BD). According to the protocol, the treated cells were harvested, collected and washed twice using phosphate-buffered saline. In a dark room, approximately 1 × 10^6^ cells in 100 μl of phosphate-buffered saline were incubated with a phycoerythrin (PE)-conjugated anti-DR4 antibody (#307206, BioLegend, San Diego, CA, USA) or negative control mouse IgG1, κ isotype antibody (#400112, BioLegend) for approximately 30 min. The gated population in flow cytometry was set on the basis of the negative control group (positive cells <1%) in every experiment. DR4 surface expression was estimated by examining the difference between the values (percentage of positive cells) of PE-DR4 and its corresponding IgG-PE control from at least three experiments in the same group.

### Statistical analysis

The experiments were performed at least three times. Representative data are shown as the mean±s.e.m. Statistical significance was identified by unpaired Student’s *t*-test analysis. *P*<0.05 was considered statistically significant.
