# Supplementary material for: JWA regulates TRAIL-induced apoptosis via MARCH8-mediated DR4 ubiquitination in cisplatin-resistant gastric cancer cells
Source: Oncogenesis. 2017 Jul 3;6(7):e353–. doi: 10.1038/oncsis.2017.57 (PMC5541709; doi:10.1038/oncsis.2017.57)
Supplement: Supplementary Information [file oncsis201757x1.docx]

JWA regulates TRAIL-induced apoptosis via MARCH8-mediated DR4 ubiquitination in cisplatin-resistant gastric cancer cells

Qiang Wang, Qi Chen, Liwen Zhu, Minjuan Chen, Wenxia Xu, Sapna Panday, Zhangding Wang, Aiping Li, Oluf D. Røe, Rui Chen, Shouyu Wang, Ruiwen Zhang, Jianwei Zhou^§^

**Supplementary Figure legends:**

**S1. DR5 has no obvious effects on TRAIL sensitivity in cisplatin-resistant GC cells. (a)** BGC823 cells were transfected with different siRNAs for DR5 or a corresponding control for 48 h. The expression of DR5 in the cells was determined by western blot analysis. **(b)** BGC823/DDP cells were transfected with si-DR4 or si-DR5-2 for 48 h and then treated with 80 ng/ml TRAIL for 24 h. The levels of DR4, DR5 and cleaved-caspase-3 were determined by western blot analyses. Representative results are shown from at least three independent experiments.

**S2. JWA negatively regulates DR4 expression. (a)** Above: SGC7901 and SGC7901/DDP cells were transfected with si-JWA or Flag-JWA or a corresponding control for 48 h; DR4 surface expression was then analysed by flow cytometry. Below: Comparison of relative DR4 surface expression between different groups. Respective DR4 surface expression was estimated by the difference between the values (% positive cells) of PE-DR4 and its corresponding IgG-PE control. For the flow cytometry assay, representative results are shown as the means ± SEM from at least three independent experiments, **P*<0.05 (Student’s t-test).

**S3. JWA promotes ubiquitination of DR4 via up-regulation of MARCH8 in GC cells. (a)** Left: Results are shown for BGC823/DDP cells co-transfected with his-JWA and Flag-DR4 (WT) or Flag-DR4 (Mut K273R) for 48 h, then exposed to 50 μg/ml CHX for the indicated times. A western blot analysis was used to determine the protein stability of Flag-DR4. Right: Quantification curve of the Flag-DR4 protein level in BGC823/DDP cells. His-con (WT-DR4) vs. His-JWA (WT-DR4), ***P*<0.01, ****P*<0.001； His-JWA (Mut-DR4) vs. His-JWA (WT-DR4), **^##^***P*<0.01, **^###^***P*<0.001  **(b)** Left: BGC823/DDP and SGC7901/DDP cells were co-transfected with his-con or his-JWA with Flag-DR4 (Mut K273R) for 48 h, and the DR4 surface expression was then analysed by flow cytometry. Right: Comparison of relative DR4 surface expression between different groups. The data of DR4 surface expression was estimated by the difference between the values (% positive cells) of PE-DR4 and its corresponding IgG-PE control in the same group. Representative results are shown from three independent experiments. For the flow cytometry assay, the data are presented as the means ± SEM, *N.S: no significance* (Student’s t-test).
