# Supplementary figures and images for: JWA regulates TRAIL-induced apoptosis via MARCH8-mediated DR4 ubiquitination in cisplatin-resistant gastric cancer cells
Source: Oncogenesis. 2017 Jul 3;6(7):e353–. doi: 10.1038/oncsis.2017.57 (PMC5541709; doi:10.1038/oncsis.2017.57)

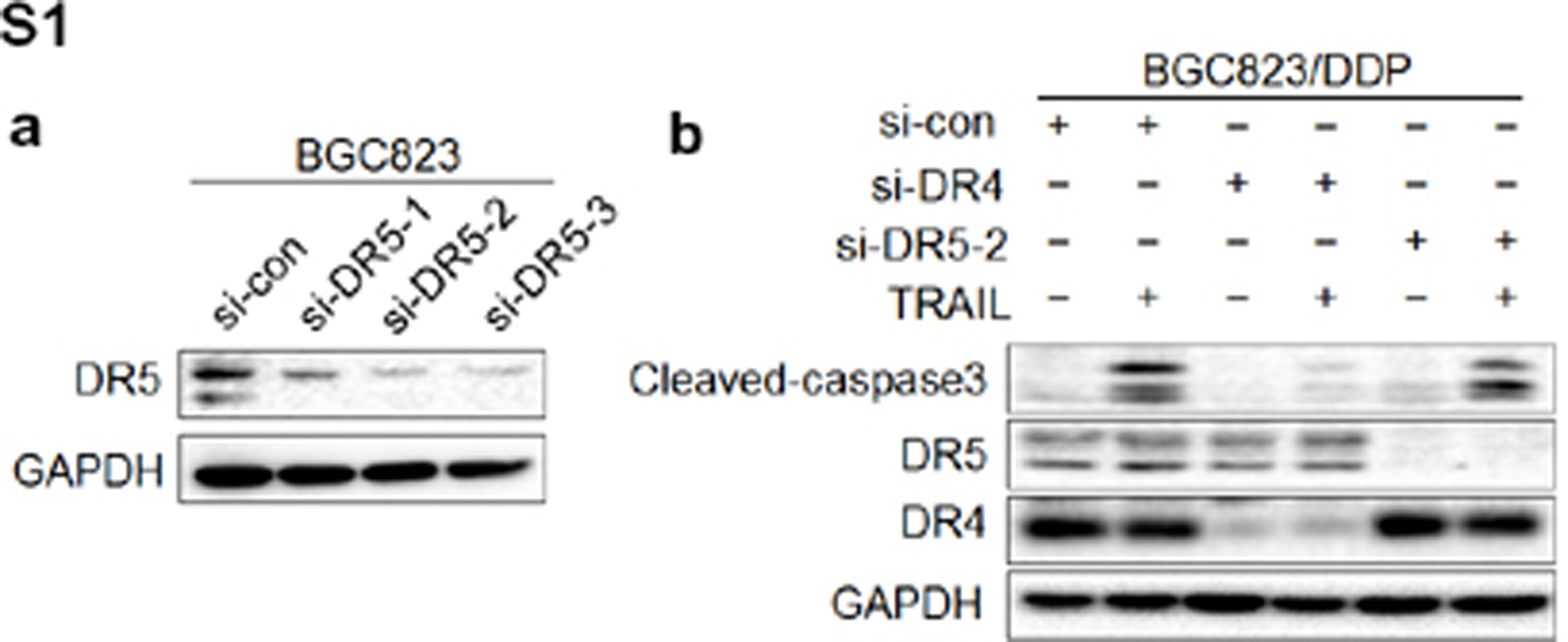

Supplement: Supplementary Figure S1 [file oncsis201757x2.tif]

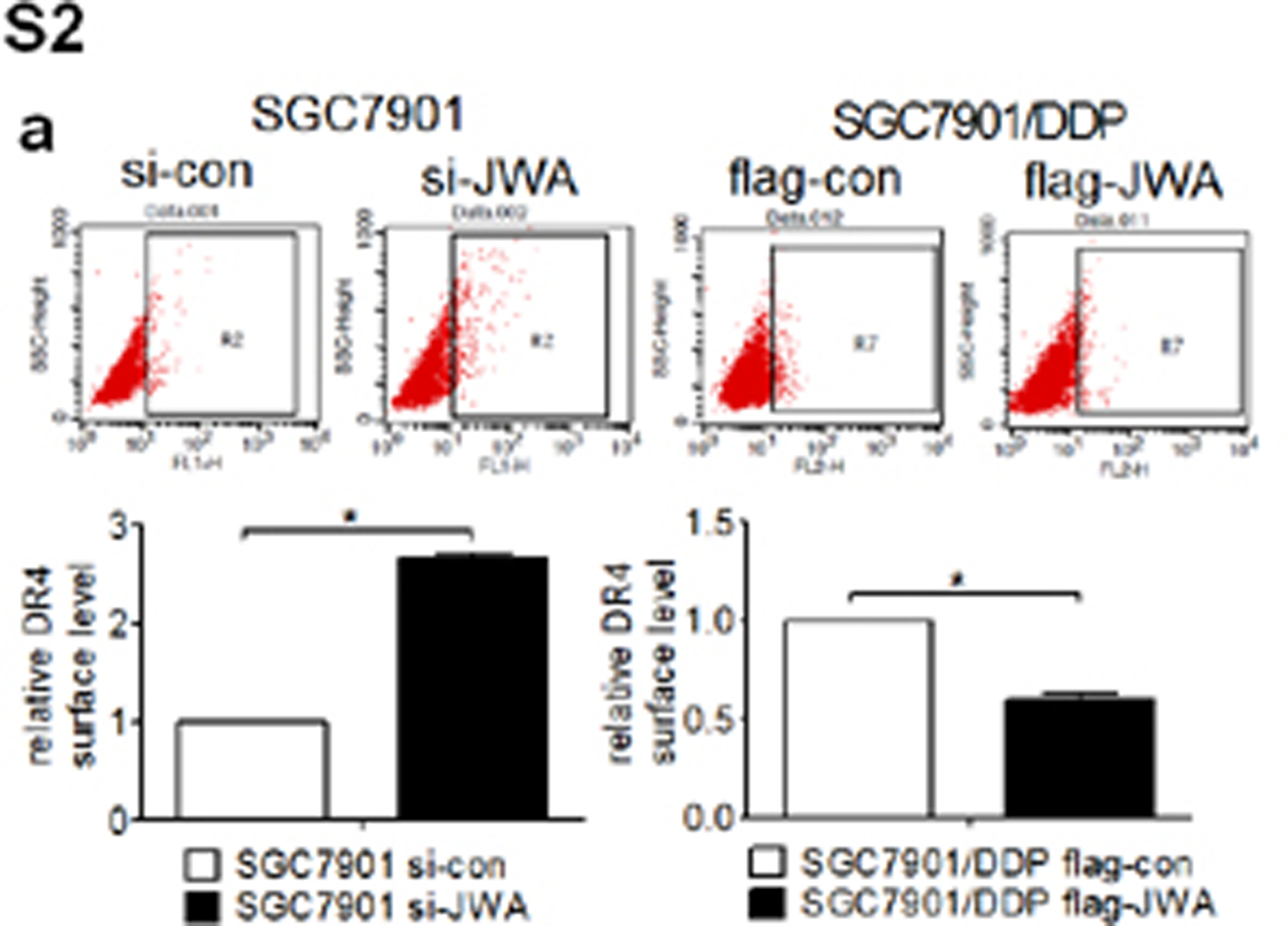

Supplement: Supplementary Figure S2 [file oncsis201757x3.tif]

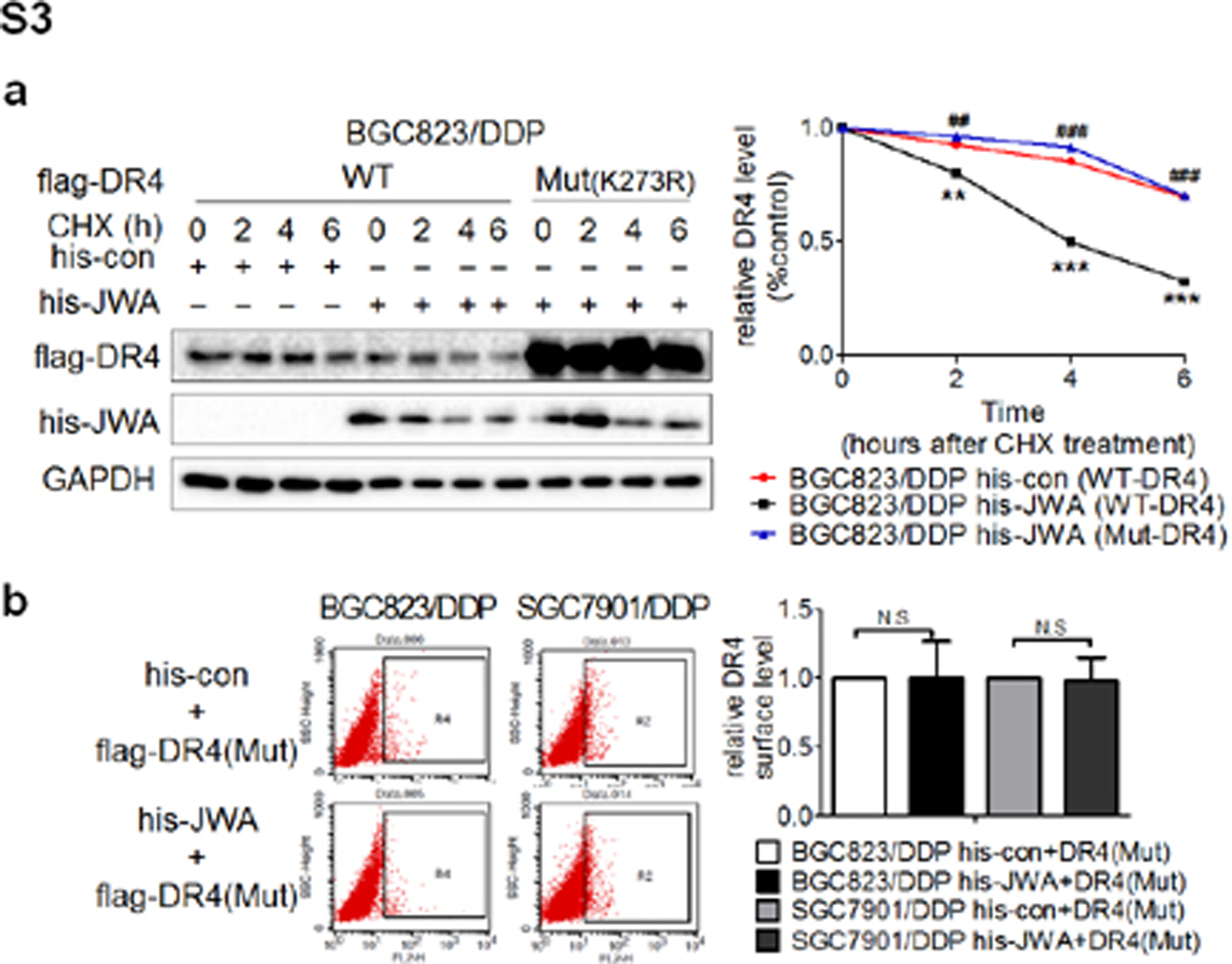

Supplement: Supplementary Figure S3 [file oncsis201757x4.tif]
